# Trends of Hepatitis A hospitalization and risk factors in Quebec, Canada, between 1990 and 2003

**DOI:** 10.1186/1471-2334-7-31

**Published:** 2007-04-18

**Authors:** Magalie Canuel, Gaston De Serres, Bernard Duval, Rodica Gilca, Philippe De Wals, Vladimir Gilca

**Affiliations:** 1 Department of Social and Preventive Medicine, Laval University, Quebec, Canada; 2 Quebec National Institute of Public Health, Quebec, Canada; 3 CHUL Research Center, CHUQ-CHUL, Quebec, Canada

## Abstract

**Background:**

In Canada, targeted vaccination of at risk groups for hepatitis A (HA) is done since the mid 1990s resulting in declining incidence. This study estimated the year and age specific hospitalization rates and distribution of risk factors for HA in Quebec, Canada, between 1990 and 2003.

**Methods:**

Records of patients hospitalized with HA-related diagnostic codes were retrieved from the provincial database. Hospital charts of all deceased cases and a random sample of all other records were reviewed.

**Results:**

From 1503 hospitalization records, 573 charts were reviewed including 49 (91%) of the 54 deceased patients. Confirmed acute HA was present in 79% of records where HA was the primary diagnosis, and in 3%–8% of records where HA was a secondary diagnosis. From the total estimated number of hospitalizations, 96% had HA as the primary diagnosis. The hospitalization rate decreased from 1.06 per 100 000 person-years between 1990 and 1997 to 0.36 between 1998 and 2003. During the study period, 54% HA hospitalizations were in 20–39 year-olds. The overall case fatality ratio among hospitalized patients was 1.4%, increasing from 0.4% in those < 40 years old to 12.5% in those ≥60 years. By decreasing order, reported risk factors were travel to HA endemic countries (30%), MSM (18%) and household contacts (11%).

**Conclusion:**

HA hospitalization rates have been low since 1998 but the cause of this is unclear given the cyclical pattern of HA. Travel to endemic countries remains the most important risk factor and improved control of HA will require better strategies to vaccinate travelers.

## Background

**Figure 1:**
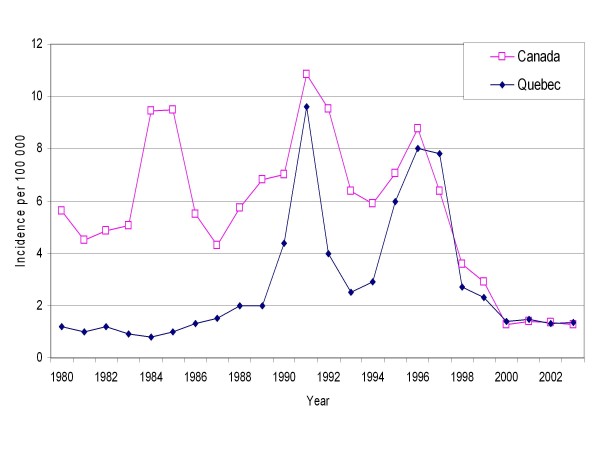
Reported Incidence per 100 000 Between 1980 and 2003 in Quebec and Canada.

The epidemiology of hepatitis A (HA) varies according to geographical, environmental and socio-economic conditions [1-3]. The reported incidence of HA in Canada remained above 4 per 100 000 between 1980 and 1997 and decreased thereafter (Figure 1) [4]. Hepatitis A exhibited a cyclical pattern with peaks in 1984, 1991 and 1996. Quebec, Canada's second largest province (7.2 million people) reported a much lower incidence before 1990 and no peak in 1984. Since 1990, incidence has been more similar to that of the country and displayed a synchrony regarding the peak years (Figure 1). These peak years occurred when two large outbreaks affected men who have sex with men (MSM) in Montreal [5,6]. After these outbreaks, the incidence declined substantially. It is hard to know if this was attributable to a larger use of the vaccine of if it is part of the normal HA cyclical pattern.

Since its licensure in 1994, HA vaccine has been offered to travelers going to HA endemic countries. However, only a small fraction consult and receive HA vaccination for which they have to pay[7,8]. Apart from the intervention targeting MSM in 1996–1997 during which 15000 doses of hepatitis A vaccine were administered free of charge to approximately 8500 individuals, there has been no further mass campaign [6]. Since 1998 in Quebec, individuals belonging to other high risk groups as defined by the National Advisory committee on Immunization (NACI) are eligible to be vaccinated free of charge [9,10]. In addition to hemophiliacs, people with chronic liver disease and household contacts of an acute case of HA, high risk groups also include MSM, illicit drug users and street youths, three groups difficult to identify and reach. While a few thousand doses are distributed annually by the regional public health units, there is currently no data regarding the overall vaccine coverage.

While there is good data on reported incidence of HA in Canada, data on hospitalization remain scarce and of uncertain validity. One study estimated hospital admissions through the Hospital Morbidity Databases from the Canadian Institute for Health Information (CIHI). It found that the age-adjusted hospitalization rate for hepatitis A decreased from 2.5 to 0.6 per 100000 persons between 1984–1998 [4]. However, only primary or principal diagnoses were analyzed and no validation was done of the diagnoses even if it is known that coding errors are present in such administrative databases [11,12]. A review of a sample of medical charts is necessary to confirm the diagnosis and obtain valid data.

In this study, we reviewed a sample of medical charts of patients recorded in an administrative database as having been hospitalized with a diagnosis of HA between 1990 and 2003 in the province of Quebec, Canada, to validate the accuracy of the diagnosis. We then estimated the age-specific rates of hospitalization and risk factors for hospitalized HA cases in that population.

## Methods

### Source of data

MED-ECHO (Maintenance et exploitation des données pour l'étude de la clientèle hospitalière) is a computerized administrative database which includes data on all admissions to one of the 136 acute care hospitals in the province. Long term care facilities are not included. A record is generated for each admission and data extracted from the hospitalization summary sheet completed by the treating physician after the patient is discharged. For each hospitalization, a primary, and up to 15 secondary diagnoses are entered, using the ninth revision of the International Classification of Diseases (ICD9). The primary diagnosis indicates the disease that lead to the admission or the complication that contributed most to the duration of the hospitalization whereas secondary diagnoses indicate underlying conditions that may or may not have contributed to the hospitalization.

### Study population

The study population included all hospitalizations between April 1^st^, 1990 and March 31^st^, 2003 for which HA was recorded at any diagnostic position. HA was identified using ICD-9 diagnostic codes 070.0 (hepatitis A with coma) and 070.1 (hepatitis A without coma). Records were excluded if the patient was not a resident of Quebec. During the study period, there was a total of 1503 records of hospitalization with HA, including 54 deaths (Table 1). HA was the primary diagnosis in 883 and one of the secondary diagnoses in 610 records.

**Table 1:** Validation of recorded HA diagnosis in hospitalized cases, Quebec, 1990–2003

	Primary Diagnosis	1^st ^secondary	2^nd ^to 15^th ^secondary¶	Total
**A – DEATHS**				
Deaths	13	3	38	54
Charts received	12 (92%)	2 (67%)	35(92%)	49 (91%)
After chart review				
Confirmed acute HA *	9 (75%)	0 (0)	2 (6%)	11 (22%)
Deaths related to HA *	8 (67%)	0 (0%)	2 (6%)	10 (20%)
**B – RANDOM SAMPLE**				
Records in MED-ECHO	880	160	409	1449
Records sampled (sampling %)	225 (26%)	110 (67%)	210 (51%)	545 (38%)
Charts received (% of requested records)	216 (96%)	103 (94%)	205 (98%)	524 (96%)
After chart review				
1)Confirmed acute HA				
caused hospitalization*	170 (79%)	8 (8%)	4 (3%)	182 (35%)
did not cause hospitalization*	1 (0.5%)	6 (6%)	11 (5%)	18 (3%)
2) No acute HA*	45 (21%)	89 (86%)	190 (93%)	324 (62%)
Prior HA	5	43	110	158
Infectious hepatitis but not A	21	17	23	61
Non-infectious hepatitis	19	21	30	70
No hepatitis	0	7	24	31
Error in data entry	0	1	3	4
**Estimated number of hospitalizations attributable to HA^†^**	**699**	**13**	**8**	**730**

* % of confirmed acute cases calculated amongst the number of charts received

^† ^Adjusted for the proportion of acute cases in 3 age groups: ≤39 years, 40–59 years, and ≥60 years.

¶ Presented in a single category as there was no difference between the different positions of diagnosis.

### Medical chart review

All 54 deaths cases and a random sample of 545 other records stratified for the position of HA diagnosis were selected for medical chart review. The random sample included 25% (225/883) of records with HA as the primary diagnosis and 56% (320/569) in any other position (Table 1). In the latter group, the sample included 67% (110/163) of cases with HA diagnosis in the 1^st ^secondary position, 52% (60/116) in the 2^nd ^secondary, 72% (60/83) in the 3^rd ^secondary and 44% (90/204) cases with HA in the 4^th ^to 15^th ^secondary positions. The sample size was calculated to provide a precision of estimates varying from ± 8% to ± 10%, assuming that the proportion of confirmed HA was 75% for diagnosis in the primary position and 20% in each secondary position.

After study participation approval by hospital's directors of professional services, archivists were provided a denominalized list of hospitalization records to be retrieved. For individual record identification, the age, gender, admission date, and diagnoses were provided to archivists by the research team. Denominalized photocopies of the medical charts for the specific hospitalization and for follow-up visits when related to HA were transmitted to the investigators. Demographic data, presence of risk factor for HA, clinical and laboratory information were extracted from the charts by study nurses on a standardized form.

### Confirmed acute HA cases

Acute HA cases were laboratory confirmed if patients had a positive IgM anti-HAV serology. Clinically confirmed acute cases were patients who did not have serological testing but had clinical signs and symptoms compatible with HA and traveled to a HA endemic country in the 60 days prior to onset of symptoms or had an epidemiological link with a laboratory confirmed HA case. Hospitalization was considered not attributable to HA if the chart clearly indicated that hospitalization was due to another disease even if HA was laboratory confirmed. Past HA cases were patients with a history of prior HA or with IgG anti-HAV testing positive but negative to or without IgM serology.

For deaths, two physicians reviewed separately the medical charts to determine if it was attributable to HA. Death was clearly attributable to hepatitis A if HA caused all or the majority of the events leading to death. Death was considered possibly attributable to HA if patients died from concurrent complications of severe underlying conditions and the role of HA was not prominent. Hepatitis A was not contributing to the death when a clearly defined etiology other than HA was the cause of the death. In the case of disagreement between physicians, they met and a consensus was reached.

### Statistical analysis

The total number of hospitalizations attributable to HA was estimated for each diagnostic position multiplying the proportion of confirmed acute HA causing the hospitalization in the random sample by the total number of records in that position plus the confirmed acute HA deceased cases. To estimate age-specific rates, this calculation was done for each age group and the result was divided by the adjusted 1996 census data used as the denominator. For the three months of 2003, rates were annualized. As two large outbreaks in the MSM community occurred in 1991 and 1995–97, the period between 1990 to 1997 was named the "outbreak period" and that between 1998 and 2003 was the "non-outbreak period". The proportion of hospitalized cases was calculated by dividing the estimated number of hospitalized cases by the number of cases reported to the Provincial Notifiable Disease Database. The case-fatality rate among hospitalized patients was calculated by dividing the number of deaths attributable to HA by the estimated total number of hospitalizations for HA. Exact binomial statistics were used to calculate 95% confidence intervals (CI). Proportions were compared using the two-sided Mantel-Haenszel χ^2 ^test or Fisher's exact test. The Wilcoxon test was used to compare non-parametrical distribution like age. Trends were evaluated with Mantel-Haenszel χ^2 ^test for trend. P value ≤ 0.05 was considered significant.

The study protocol was approved by Quebec University Hospital Center's Research Ethics Committee.

## Results

Of the 54 requested medical charts of deceased patients, 49 (91%) were obtained, whereas in the random sample, 524 (96%) of the 545 requested charts were received (Table 1). The most frequent reason for not getting a chart was the impossibility for hospital archivists to identify a patient by lack of computerized data at the beginning of the study period in some hospitals. The median age was similar in patients whose charts were reviewed and the other patients (P = 0.55) when the diagnosis was in primary position but it was 4 years younger when the diagnosis was in any secondary position (P = 0.002).

The chart review of the 49 deceased patients found that only 11 (22%) had a confirmed acute HA at the time of hospitalization. The proportion of confirmed acute cases was 67% (9/12) when HA was the primary diagnosis in contrast to 5% when HA was a secondary diagnosis (Table 1). Amongst these 11 confirmed acute cases, one died from an apparently unrelated pneumonia whereas death was attributable to hepatitis A virus for the other 10 (7 clearly, 3 possibly) cases. The age distribution of these 10 cases was 2 patients < 40 years old, 4 aged 40–59 years and 4 who were ≥60 years. Three had no underlying medical conditions, three had a chronic hepatitis B, one had alcoholic cirrhosis and 3 had a cardio-vascular disease.

In the random sample, the review of the 524 medical charts showed that 324 (62%) patients did not have acute HA, 182 (35%) were hospitalized for confirmed acute HA (157 (86%) were laboratory confirmed) and 18 (3%) had acute HA that did not cause the hospitalization. Of the 26 (14%) HA cases without serology testing, 16(62%) occurred before 1994 and only 1 (3%) had no testing in 1999–2003. Among patients with no acute HA, 158 (49%) had a history or a serologic evidence of past HA, 131 (40%) had an acute hepatitis of another etiology, and 35 (11%) had no hepatitis at all (Table 1). Among the 182 confirmed cases, 7 (4%) had a chronic liver disease. No liver graft was reported in any of the reviewed charts.

In charts where HA was the primary diagnosis, 79% were confirmed acute cases. This proportion was 82% for the patients <60 years and 42% for those 60 years and older (P = 0.003). Overall, the proportion of confirmed acute HA cases was 8% for a diagnosis in 1^st ^secondary and 3% for other secondary diagnosis (Table 1) with no statistically significant difference between age groups for these positions (P ≥ 0.08). The total estimated number of hospitalizations attributable to HA during the study period was 730 and 96% of them had HA as the primary diagnosis. As 522 (72%) cases were <40 years of age, 176 (24%) were 40–59 years and 32 (4%) were ≥60 years old, the estimated case-fatality rate among hospitalized patients was 1.4% (CI 95% 0.66%–2.50%), increasing from 0.4% before 40 years of age (CI 95%, 0.05%–1.37%), to 2.3% (CI 95%, 0.62%–5.70%) for those between 40 and 59 years, and to 12.5% (CI, 3.5%–29.0%) for those ≥60 years of age.

**Figure 2:**
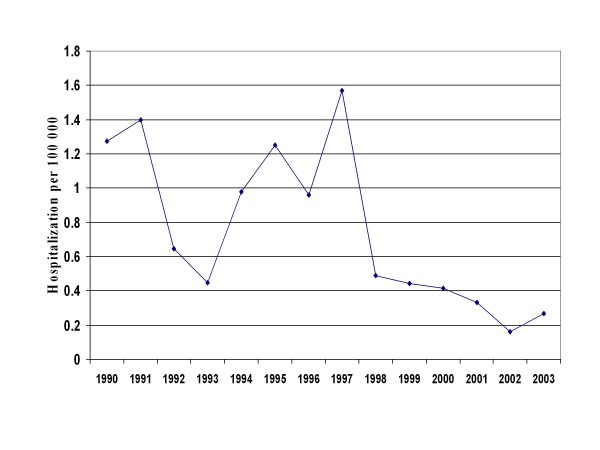
Hepatitis A Hospitalization Rate per 100 000 Between 1990 and 2003 in Quebec, Canada.

Adjusting the number of hospitalizations reported in MED-ECHO with the percentage of confirmed acute cases found in the chart review, the mean hospitalization rate for the study period was estimated at 0.77 per 100000 persons, decreasing from 1.06 per 100000 persons during the outbreak period to 0.36 in the non-outbreak period (Figure 2). For the study period, the hospitalization rate was 0.7, 1.15, 0.68 and 0.21 per 100000 among persons aged less than 20 years, 20–39 years, 40–59 years and 60 years and older respectively. The mean hospitalization rate per 100 000 for the outbreak and non-outbreak period was 0.86 and 0.53 respectively for the patients aged less than 20 years, 1.68 and 0.35 for the 20–39 years old, 0.97 and 0.34 for the 40–59 years old and 0.27 and 0.09 for the 60 years and older. Overall, the median length of stay for HA was 4 days: it increased from 3 days for patients aged less than 20 years to 4, 5 and 7.5 days for those aged 20–39, 40–59 and 60 years and older respectively (χ^2 ^for trend, P = 0.05). Overall, 19% of hospitalizations occurred in those less than 20 years, 54% in 20–39 year-olds, 23% in 40–59 year-olds, and 4% in 60 years and older (p < 0.001). For the entire study period, 19% of cases reported to the Provincial Notifiable Disease Database were hospitalized. It was 21% in patients aged less than 20 years, 17% for those 20–39 years, 23% for those 40–59 years and 16% for patients 60 years and older (P = 0.7).

Epidemiological risk factors for acquisition of HA were present in the medical charts of 119 (65%) of the 182 confirmed acute cases. By order of declining importance, non-mutually exclusive risk factors were: travel to endemic countries (30%), MSM (18%), household contact of a case (10%), IDU (6%) and others (homeless, institutionalized patient, patient with chronic liver disease) (5%). MSM represented 22% of hospitalized cases in the outbreak period and 5% in the non-outbreak period whereas travel to a HA endemic country was almost unchanged during the two periods (30% vs 33%).

## Discussion and conclusion

Administrative hospitalization databases can be helpful in identifying patients hospitalized for a specific condition and are used to evaluate the epidemiology, the burden of the disease, and the impact of a vaccination program [12-15]. As diagnoses recorded in MED-ECHO are written by the treating physician on the hospital separation sheet, the results of this study are likely to apply to similar administrative databases relying on the physicians' diagnoses. As 96% of the total estimated number of hospitalized HA cases had their diagnosis in the primary position in our study, this suggests that focusing a search for acute cases of HA among those hospitalized with a diagnosis in the primary position in similar administrative database would not miss many cases. However, diagnosis must be confirmed to ensure the validity of results as demonstrated by the review of medical charts which found confirmation of HA in 75% of patients who died and in 79% of other patients. Thus, non validated estimates as those calculated for Canada using only the principal or primary diagnosis might overestimate HA hospitalization rates by 20% to 25% [4]. Thus the overall hospitalization rate of 1.06 per 100 000 estimated in Quebec for the outbreak period (1990–1997) compared to 1.26 reported for Canada suggest that the actual rates were similar.

However both estimates limited the case ascertainment to records where HA was diagnosed, a strategy which may underestimate the hospitalization rates. We did not seek cases among patients with other diagnoses such as hepatitis B, other hepatitis, or liver disease. Patients whose medical charts had no laboratory results and no data concerning possible epidemiological links as undetermined hepatitis, but may in fact have been HA cases. This is unlikely to have significantly biased the results as it occurred only in 4% of the primary diagnosis and did not happen for any other diagnosis position. Also, given the very low percent of confirmed acute cases in patients where HA was recorded as a secondary diagnosis it is expected that this proportion would be much lower in patients where HA is not a recorded diagnosis.

Almost half of the confirmed HA cases with death occurred in persons aged 60 years and older and our case-fatality ratio was highest in this age group. This is consistent with previous data which showed that severity of the disease and case-fatality rates increase with age[3,16]. Our overall proportion of incident HA cases that were hospitalized was 19% which is in the range of proportion reported in the USA (11–22%)[17]. Our rates of hospitalization estimated for the period 1998–2003 are lower than those from England and Wales, Belgium, Austria and Germany (> 0.8 per 100 000) but higher than in the Netherlands (≤ 0.2 per 100 000)[18]. There is no recent data on HA hospitalization rate in the USA, but combining the number of hospitalized cases of HA from the hepatitis surveillance report in 2002 [19] and data from the Bureau of Census Estimates of the U.S Residents [20], in 2002 it can be estimated to be in the range of 0.31 to 0.36 per 100000, similar to our estimates.

While it is possible that risk factors differ between non-hospitalized and hospitalized HA cases, hospitalized cases are likely to give a good representation of risk factors existing in the population. The outbreaks in MSM in the 1990s evidenced that they constitute a group where long lasting transmission can occur and their vaccination remains a priority. However, even during the MSM outbreak years, travel to an HA endemic country was the most frequently reported risk factor and has been present in 32% of hospitalized cases since 1998 (data not shown). Vaccination of these travelers is unlikely to improve rapidly with the current travel clinic strategy and importations of HA will probably continue [7,8]. The cause of the lower incidence and hospitalization rates observed in Quebec since 1998 is still unclear but is also similar to those observed in other low endemicity countries. Given the cyclical pattern of HA and because the vast majority of the population remains unvaccinated, we cannot rule out a resurgence of this disease in the next years despite some use of the vaccine by individuals belonging to high risk groups.

## Competing interests

MC, RG and VG-none; GDS, BD and PDW served as unpaid members of vaccine advisory committee, GSK Canada, and have received research grants from GSK.

## Authors' contributions

MC did the acquisition, analysis and interpretation of data and drafted the manuscript. GDS did the conception and design of the study and participated to the acquisition, analysis and interpretation of data and revised the manuscript. BD participated to the interpretation of data and revised the manuscript. VG participated to the analysis and interpretation of data and revised the manuscript. RG and PDW revised the manuscript. All authors read and approved the final manuscript.

## Pre-publication history

The pre-publication history for this paper can be accessed here:


